# 4141 Molecular Signatures of Cocaine Toxicity in Postmortem Human Brain and Neurons

**DOI:** 10.1017/cts.2020.414

**Published:** 2020-07-29

**Authors:** Emily Frances Mendez, Laura Stertz, Gabriel Fries, Ruifeng Hu, Thomas Meyer, Zhongming Zhao, Consuelo Walss-Bass

**Affiliations:** 1 The University of Texas Health Science Center at Houston

## Abstract

OBJECTIVES/GOALS: The goal of this project is to identify new therapeutic targets and biomarkers to treat or prevent cocaine toxicity by investigating proteomic, transcriptomic and epigenetic signatures of cocaine exposure in human subjects. METHODS/STUDY POPULATION: Cocaine is a highly addictive neurotoxic substance, and it is estimated that 1.9 million Americans are current users of cocaine. To study the molecular effects of cocaine, we generated preliminary proteomics and next-generation RNA sequencing (RNAseq) data from human postmortem dorsolateral prefrontal cortex (Broadmann area 9 or BA9) of 12 cocaine-exposed subjects and 17 controls. Future directions for this project include RNAseq and DNA methylation analysis of neuronal nuclei sorted from human postmortem BA9 and a human induced pluripotent stem cell-derived neuron (hiPSN) model of cocaine exposure from the same postmortem subjects from whom we have brain samples. RESULTS/ANTICIPATED RESULTS: We found alterations in neuronal synaptic protein levels and gene expression, including the serotonin transporter SLC6A4, and synaptic proteins SNAP25, SYN2, SYNGR3. Pathway analysis of our results revealed alterations in specific pathways involved with neuronal function including voltage-gated calcium channels, and GABA receptor signaling. In the future, we expect to see an enhancement in neuron-specific gene expression signatures in our sorted neuronal nuclei and our hiPSN model of cocaine exposure. The hiPSN model will help elucidate which effects are due to acute versus chronic exposure of cocaine. DISCUSSION/SIGNIFICANCE OF IMPACT: Neuronal signatures found with this analysis can help us understand mechanisms of cognitive decline in long-term cocaine users as well as the acute effects on the brain of cocaine taken in overdose. With this work and future proposed studies, we can discover novel clinical biomarkers for cocaine neurotoxicity in patients with cocaine use disorder and determine readouts for future therapeutic development on cocaine addiction and overdose.

